# Carbapenem-Resistant *Serratia marcescens*: Genomic Plasticity, Virulence Architecture, and the Expanding Threat of Multidrug Resistance

**DOI:** 10.3390/antibiotics15040359

**Published:** 2026-04-01

**Authors:** Theodoros Karampatakis, Katerina Tsergouli, Payam Behzadi

**Affiliations:** 1 Department of Clinical Microbiology, University Hospital Kerry, V92 NX94 Tralee, Ireland; 2 Department of Microbiology, Shahr-e-Qods Branch, Islamic Azad University, Shahr-e Qods 37541-374, Iran

**Keywords:** *Serratia marcescens*, pangenome, virulence factors, bacterial drug resistance, carbapenem resistance, horizontal gene transfer

## Abstract

*Serratia marcescens* is a highly adaptable Gammaproteobacterium with broad ecological distribution and growing clinical importance. Advances in whole-genome sequencing (WGS) and pangenome analysis reveal extensive genomic plasticity, driven by mobile genetic elements (MGEs) such as plasmids, transposons, integrons, prophages, and extracellular vesicles, which collectively accelerate virulence and antimicrobial resistance (AMR) evolution. *S. marcescens* displays a dynamic accessory genome enriched in resistance and virulence determinants, supporting persistence in diverse environments, including hospital water systems. Clinically, *S. marcescens* is an emerging opportunistic pathogen associated with severe healthcare-associated infections, ICU outbreaks, and multidrug-resistant “superbug” phenotypes. Its resistome includes intrinsic AmpC β-lactamase, broad efflux systems, and chromosomal determinants conferring resistance to β-lactams, polymyxins, and multiple additional drug classes, while acquired ESBLs and carbapenemases urther limit therapeutic options. Integrating genomic, evolutionary, and clinical insights underscores the urgent need for improved surveillance, mechanistic understanding, and targeted interventions against carbapenem-resistant *S. marcescens* (CRSM).

## 1. Introduction

*Serratia marcescens* is a rod-shaped, ubiquitous Gram-negative bacterium known for producing the red pigment prodigiosin, which can resemble blood on starchy substrates [1,2,3,4,5,6]. This striking feature historically led to cultural and religious interpretations, such as the 1263 Mass in Bolsena, later explained by Bartholomeo Bizio as microbial in origin in the nineteenth century [3,4,5,6]. Although the term “miracle bacterium” is now obsolete, the pigmentation of *S. marcescens* remains a notable example of the intersection between microbiology and cultural perception [3,4,5,6].

### 1.1. Taxonomy

In accordance with the List of Prokaryotic names with Standing in Nomenclature (https://lpsn.dsmz.de/genus/serratia (accessed on 12 February 2026)), the genus *Serratia* comprises 24 validly published species, among which *S. marcescens* is a recognised member [7]. According to LPSN and NCBI/Taxonomy [https://www.ncbi.nlm.nih.gov/datasets/taxonomy/tree/ (accessed on 12 February 2026)], *Serratia* belongs to kingdom Pseudomonadati, phylum *Pseudomonadota*, class *Gammaproteobacteria*, order *Enterobacterales*, and family *Yersiniaceae* (https://www.ncbi.nlm.nih.gov/datasets/taxonomy/615/ (accessed on 12 February 2026) [8].

Since 2016, *Enterobacterales* has been reorganised into eight valid families—*Budviciaceae*, *Enterobacteriaceae*, *Erwiniaceae*, *Gallaecimonadaceae*, *Hafniaceae*, *Morganellaceae*, *Pectobacteriaceae*, and *Yersiniaceae*. Under this revised taxonomy, genera such as *Serratia* (*Yersiniaceae*), *Edwardsiella* and *Hafnia* (*Hafniaceae*), *Morganella*, *Proteus*, and *Providencia* (*Morganellaceae*), and *Yersinia* (*Yersiniaceae*) have been removed from the traditional *Enterobacteriaceae* family [8,9,10].

*Enterobacterales* are Gram-negative, rod-shaped, non-spore-forming facultative anaerobes whose evolutionary distinction is defined by five conserved insertion–deletion mutations in genes encoding L-arabinose isomerase, an EF-P-like protein (YeiP), a hypothetical protein, a peptide ABC transporter permease, and pyrophosphatase [8]. Hypothetical proteins are in silico predictions produced in dry labs using bioinformatic software tools but lack confirmation through wet lab experiments. Computational analyses, including Basic Local Alignment Search Tool [BLAST, https://blast.ncbi.nlm.nih.gov/Blast.cgi, v. 2.17.0 (accessed on 10 February 2026)] and structural modelling, help infer their potential functions and underscore their evolutionary and therapeutic importance. BLAST identifies regions of sequence similarity to support such predictions [11,12,13,14,15,16,17,18,19].

Notably, *Serratia* has also been traditionally placed within *Enterobacteriaceae* due to its phenotypic characteristics [2]. Although the genus comprises several species, research has largely focused on the type species *S. marcescens* because of its value as a model organism and its growing relevance in healthcare-associated infections (HAIs) [1,2].

*S. marcescens* has been central to studies on motility and secretion systems and is increasingly recognised as a clinically important opportunistic pathogen, marked by rising multidrug resistance and challenging nosocomial infection profiles [2,20,21,22].

### 1.2. Carbapenem-Resistant Serratia marcescens (CRSM)

*S. marcescens* is a highly adaptable bacterium capable of surviving in a wide range of environments, from natural habitats to healthcare settings [20,23]. Its remarkable stress tolerance enables colonisation of medical surfaces, facilitating healthcare-associated transmission. This opportunistic pathogen causes diverse infections, including arthritis, endocarditis, pneumonia, meningitis, osteomyelitis, and skin infections, urinary tract infections, keratitis, wound infections, peritonitis, sepsis and bacteremia, posing significant risks to immunologically vulnerable patients, particularly those in intensive care units (ICUs) [23,24,25,26,27,28,29,30,31]. *S. marcescens* contributes to ICU outbreaks and the spread of carbapenem-resistance, leading to the emergence of carbapenem-resistant *S. marcescens* (CRSM) [32]. As an ESCPM group member (including *Enterobacter*, *Serratia*, *Citrobacter*, *Providencia*, and *Morganella*), it harbours a chromosomal AmpC β-lactamase conferring intrinsic β-lactam resistance, which can be amplified during therapy [32,33,34]. The emergence of plasmid-mediated Extended Spectrum β-lactamases (ESBLs) and carbapenem resistance has further limited treatment options [32]. ESBLs, which have evolved from standard β-lactamases via mutation, are mostly classified as Ambler class A β-lactamases (e.g., CTX-M/TEM/SHV). Although they are derivatives, the ESBL phenotype can also be seen with some class D OXA variants. These enzymes are structurally characterised by a serine residue at their active site. Accordingly, they are referred to as serine-based β-lactamases (SBLs) [35,36,37].

Multidrug-resistant (MDR) *S. marcescens* and CRSM strains cause severe healthcare-associated infections, particularly in pediatric and ICU patients [32,34,38]. AMR is classified by its extent: MDR (strains are resistant to at least one drug in three or more antimicrobial categories); extensively drug-resistant (XDR) (strains retain susceptibility to only one or two categories); and pan drug-resistant (PDR) (strains are resistant to all available antimicrobial agents) [37,39,40,41,42,43,44]. The emergence of MDR bacteria, or “superbugs”, poses a critical global health threat [45,46,47]. Given the multifaceted significance of *S. marcescens* and CRSM, this study aims to investigate CRSM-specific mobilome, resistome, and virulome synthesis, high-risk genes and mobile genetic elements (MGEs).

## 2. Genomic Pool, Pangenome and Genomic Plasticity of *Serratia marcescens*

Advances in genomic sequencing have shifted bacterial classification from phenotypic criteria to genotype-based approaches [48]. High-throughput technologies and whole-genome sequencing (WGS) allow comprehensive characterisation of microbial genomes, while pangenome analysis provides a framework for interpreting genomic diversity [48]. A species’ pangenome generally consists of three components: the core (or persistent) genome shared by nearly all strains; the accessory (or adaptive/flexible/dispensable) genome present in many but not all strains and contributing to phylogroup-specific variation; and the rare (or unique/singleton/cloud) genome comprising genes found in only one or a few strains [40,43,44,48,49,50]. The core—or persistent—genome predominantly comprises housekeeping and metabolic genes, along with a small proportion of transposable elements (TEs) and genes of unknown function. In contrast, the adaptive genome includes a diverse set of genes with broad functional roles, as well as both characterised and uncharacterized mobile genetic elements (MGEs), or the mobilome, such as plasmids, integrons, transposons, and insertion sequences (ISs) [40,43,44,48,50,51]. The cloud genome is enriched in TEs (e.g., transposons, ISs, Integrons), which serve as vehicles for passenger or cargo or jumping genes. In this context, horizontal gene transfer (HGT) —through conjugation, transformation, transduction, and vesiduction—plays a central role in the movement of genomic elements (GEs) and genomic islands (GIs) [40,43,44,48,50,51]. Conjugation is a major leading power of plasmid-mediated ARG dissemination in some bacterial groups like *Enterobacterales*. Plasmids in Gram-negative bacteria fall into 27 well-established incompatibility groups, with IncA/C, F, H, L/M, and P widely distributed and commonly ARG-bearing [52,53]. Yet clinically important genes—including ESBLs, carbapenemases, 16S methyltransferases, and *mcr*—are not restricted to specific plasmid groups [52,53]. Moreover, the mobility of plasmids across microbial species creates a dynamic, moving target that significantly complicates the tracking and management of drug-resistant bacteria [53].

Natural transformation occurs in two stages: a type IV pilus captures environmental DNA (e-DNA), which binds surface proteins such as ComEA. One strand enters through ComEC while the other is degraded, and the resulting ssDNA, potentially resistant to restriction, integrates via RecA-mediated homologous recombination [53]. Godeux et al. [53,54] showed that Gram-negative bacteria, including *A. baumannii*, use transformation to transfer large GEs, such as AbaR4 carrying *bla*_OXA-23_ and AbaR1, which harbours multiple resistance genes, including *aac(3)*, *aph(3′)*, *aad* variants, *cat*, *tetA*, *bla*_OXA-10_, and *bla*_PRE-1_ [53,54].

Transduction transfers DNA between bacteria via bacteriophages, occurring as generalised or specialised. In addition to phage genes, ARGs—including *bla*_TEM-1_, *armA*, *aac*, *aph*, *aad*, *mph*, and *tetA*—and intrinsic resistance determinants like *gyrA* (S81L) can be mobilised, often along with up to 35 kb of adjacent genomic DNA [53,55,56,57,58]. Phages carrying ARGs, including *bla*_TEM_ and *bla*_CTX-M_, are found in hospital effluents, wastewater, and rivers, and can transfer these genes to *E. coli*, demonstrating transduction’s environmental role in spreading AMR [53,59,60,61].

AMR can spread via the fourth HGT mechanism of vesiculation, where extracellular vesicles (EVs) transfer plasmids and resistance genes between bacteria, rapidly disseminating β-lactamase and carbapenemase genes (e.g., *bla*_CTX-M_, *bla*_KPC_, *bla*_OXA_, *bla*_NDM_) across species like ESBL-producing *E. coli* and carbapenem-resistant *K. pneumoniae* (CRKP) [43,44,58,62,63,64,65,66,67,68,69,70]. Antibiotic exposure further enhances EV-mediated transfer, accelerating AMR spread in clinical and environmental settings. EVs also carry proteins, lipids, nucleic acids, and pathogenic virulence factors, highlighting their broad role in bacterial adaptation and pathogenicity [43,44,62,63,64,65,66,67,68,69].

Previous studies have shown that members of the *Tn3* family are highly effective transposons involved in the dissemination of AMR features, particularly carbapenemase genes and colistin resistance genes, among *Enterobacteriaceae* [51,71,72]. Genomic analyses of *S. marcescens* reveal well-defined phylogenetic lineages, many of which correlate with specific infection sources. These associations suggest niche-adapted evolutionary trajectories, further supported by the distinctive accessory genome profiles characterising each lineage [1,2,31,73,74].

According to the NCBI Genome database, 3864 genomes from various *S. marcescens* isolates are currently available (https://www.ncbi.nlm.nih.gov/datasets/genome/?taxon=615 (accessed on 12 February 2026)).

Among these, ASM3029173v1 is designated as the reference genome (RefSeq). This genome has a total size of 5.2 Mb and consists of two circular replicons: a plasmid (pELP1.10) measuring 129,237 bp and a chromosome measuring 5,031,539 bp. The assembly contains 4906 genes, including 4752 protein-coding genes and coding sequences (CDSs). The genomic GC content is 59.5%. This strain was isolated from soil in Telegraph Bay, Hong Kong [https://www.ncbi.nlm.nih.gov/datasets/genome/GCF_030291735.1/ (accessed on 12 February 2026)].

Among *Serratia* species, genome sizes vary considerably [https://www.ncbi.nlm.nih.gov/datasets/genome/?taxon=613 (accessed on 12 February 2026)]. *S. fonticola* possesses the largest genome, approximately 6.0 Mb with a single chromosome and 5440 predicted genes [https://www.ncbi.nlm.nih.gov/datasets/genome/GCF_001006005.1/ (accessed on 12 February 2026)], whereas *S. microhaemolytica* has the smallest genome at about 3.3 Mb, comprising 2889 genes [https://www.ncbi.nlm.nih.gov/datasets/genome/GCF_004011885.1/ (accessed on 12 February 2026)].

Nuncio-García et al. [75] described the genome of *S. marcescens* CH31, isolated from *Periplaneta americana* (American cockroach) near a tertiary hospital in Mexico. The strain carries a single chromosome and one plasmid, totalling 5.63 Mb with a GC content of 58.96% [75]. These features exemplify the genus’s notable genomic plasticity, which underpins its pathogenicity potential and capacity for AMR gene dissemination [76,77,78].

A bacterial pangenome encompasses all genes within a species, though strain-to-strain diversity can be substantial. Some taxa exhibit highly fluid genomes enriched in accessory genes associated with traits such as virulence or AMR, whereas others retain a more conserved core genome. Evidence suggests that an ecological lifestyle is a major determinant of this variability [79]. Dewar et al. [79] further showed that host-associated bacteria, including pathogens, less-motile forms, intracellular species, and obligate symbionts, display significantly lower pangenome fluidity than free-living bacteria, a pattern driven by lifestyle rather than effective population size. Thus, Dewar et al. [79] demonstrated that bacterial lifestyle shapes both overall genome architecture and the inter-strain diversity that underlie pangenome structure. *Serratia* species are highly adaptable and occupy diverse environments, with aquatic habitats (both in nature and hospital water systems) being especially common [2,20,80]. While *S. rubidaea* and *S. liquefaciens* are occasionally associated with HAIs [78,81], many *Serratia* species are entomopathogenic, including *S. proteamaculans* and *S. marcescens* [25,82]. Notably, *S. marcescens* bridges ecological and clinical relevance and dominates available genomic datasets, including those from metagenomic studies of preterm infants and hospital environments [73]. Furthermore, *S. ficaria* participates in the fig wasp mutualism, highlighting the genus’s ecological breadth. Although it can be isolated from various clinical specimens, it rarely causes severe disease, and most infections remain mild [83,84,85,86].

As proposed by Ramdass and Rampersad [77], the metabolic plasticity of *S. marcescens* represents an adaptive evolutionary response to persistent environmental pressures.

Ono et al. [31] updated the *S. marcescens* complex by analysing more than 200 newly sequenced isolates, resolving 14 phylogenetic clades shaped by MGEs acquisition, e.g., plasmids, prophages and integrase genes. MDR strains were restricted to clades 1 and 2, dominated by hospital-associated isolates carrying diverse ARGs, including ESBL-encoding genes, carbapenemase genes, and fluoroquinolone resistance (FQR) mutations [31]. These findings reported by Ono et al. [31] re-evaluate the species’ evolutionary structure and pinpoint the clades of greatest clinical concern.

Sanchez-Perez et al. [87] showed that three pigmented *S. marcescens* isolates (HU1848, HU2225, HU2228), though phylogenetically aligned with environmental strains, carry multiple virulence factors and ARGs to chloramphenicol, fosfomycin, and tetracycline, yet remain susceptible to aminoglycosides and fluoroquinolones. Methyl gallate affected pigment production and motility, and the absence of N-Acyl Homoserine Lactone (AHL) biosynthetic genes contradicted the assumed quorum-sensing (QS) control of prodigiosin synthesis [87]. In *Serratia* spp., AHLs regulate diverse phenotypes, including virulence factors (e.g., production of enzymes, antibiotics, and prodigiosin), motility, pathogenicity, biosurfactant production, biofilm formation, and 2,3-butanediol fermentation [88,89,90]. Sanchez-Perez et al. [87] indicated that environmental lineages can persist in clinical settings and may contribute to HAIs.

Abreo and Altier [74] assessed genotype–source associations in *S. marcescens* by analysing 45 strains within a pangenome framework. Their 19,469-gene pangenome comprised 84% flexible or cloud genes and only 16% core genes, highlighting the species’ substantial accessory repertoire and well-documented ecological adaptability [74].

Robust methodologies and reliable pangenome analyses support a shift from traditional and classical Darwinian evolutionary perspectives to a Woeseian, a term denoting the model introduced by Carl Richard Woese, the HGT-driven model of microbial evolution, thereby improving diagnostic accuracy and treatment selection [48].

As noted above, the pronounced genomic fluidity and plasticity of *Serratia* spp., including *S. marcescens*, endow this species with a broad and dynamic repertoire of virulence determinants (virulome) and ARGs (resistome).

## 3. Virulence Factors and Strategies

**Figure 1:**
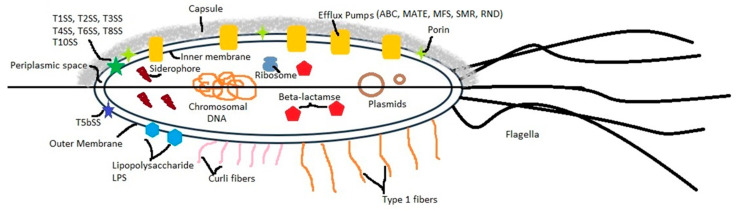
*Serratia marcescens* and its virulome and resistome.

*S. marcescens* is a major contributor to HAIs and is routinely identified among the most concerning hospital pathogens. Its pathogenic capacity stems from a diverse suite of virulence determinants, such as flagella, proteases, and hemolysins, along with an unusual ability to withstand medical solutions and disinfectants, facilitating persistent contamination and recurrent clinical outbreaks [91,92,93,94,95,96,97]. To persist in diverse environments and host tissues, *S. marcescens* deploys adhesion factors and multiple secretion systems that enable tissue invasion and immune evasion. The cooperative action of these mechanisms greatly amplifies its pathogenic potential. The virulome of *S. marcescens* (Figure 1) is composed of a wide range of pathogenic strategies including adhesion [adhesion-like curli-like fibers, type 1 fibers [98], capsule and outer membrane proteins (OMPs)], hemolysis (e.g., hemolysins and cytotoxins), extracellular enzymes (such as nucleases and DNases, proteases (including serralysin), lipases and phospholipases), prodigiosin pigment production, secretion systems, iron acquisition systems (such as siderophores, QS and regulation.

- **Curli-like fibers:** Research on *S. marcescens* pili and fimbriae remains limited. Campos et al. [99] reported thin fibrillar surface structures, and Boldeanu et al. [20] proposed an adhesive role, though mechanisms were not defined. In contrast, curli fimbriae (amyloid fibres produced via a dedicated assembly pathway) are well characterised in *Enterobacteriaceae* (e.g., *E. coli*) and are known to mediate surface adhesion and promote robust biofilm formation [42,100]. Indeed, curli fibres are produced via a nucleation–precipitation pathway, which is classified as the Type VIII secretion system (T8SS) [101].
- **Type 1 fibers:** Chaperone–Usher (CU) fimbriae, key adhesion and biofilm mediators in *Enterobacteriaceae*, are poorly characterised in *S. marcescens* [42,98,100,102,103,104,105,106,107,108]. Readily metabolised carbon sources activate cAMP–CRP-dependent catabolite repression, which suppresses Type 1 fimbrial expression in *S. marcescens* and *Vibrio cholerae*, thereby reducing biofilm formation. In contrast, the same regulatory system can enhance biofilm development in other species, including *E. coli* and *P. aeruginosa* [109,110]. Gonzalez-Montalvo et al. [98] identified 421 fimbrial usher proteins (FUPs) across 39 *S. marcescens* genomes, defining 20 CU operons, six forming the core fimbriome (Fgov, Fgfo, Fgft, Fpo, Fps, and Fso; detected among ≥94% of the *S. marcescens* strains). Three core operons (*fgov*, *fpo*, *fps*) are primarily expressed and act as major adhesins, with Fgov (former Fim) mediating yeast agglutination, human corneal epithelial (HCE) cell adhesion, and biofilm formation [98,111,112]. Core fimbriae provide general adhesion (known as housekeeping adhesins), while the strain-specific repertoire drives niche adaptation, phenotypic diversity, and genomic plasticity [74,77,79,98].
- **Capsule:** Capsule production is a conserved virulence trait among *Enterobacteriaceae*, providing effective protection against host immune clearance. In *S. marcescens*, the capsule is composed of acidic polysaccharides, with its chemical composition varying across clinical isolates. Notably, capsule expression is considered essential for the establishment of bloodstream infections. Thus, the polysaccharide capsule is known as a virulence factor in *S. marcescens* [113,114,115,116,117].

Acidic polysaccharides constitute the *S. marcescens* capsule, although their composition varies among isolates [114]. A defining feature of *S. marcescens* is the absence of *wzi* from its capsule locus, though a distant homolog exists. As an outer membrane anchor, *wzi* differentiates cell-associated CPS loci from secreted exopolysaccharides like colanic acid [113,118,119]. Biochemical analyses further separate neutral O antigens from acidic CPS, the latter consistently containing glucuronic and galacturonic acids across K serotypes but absent from O-antigen structures [114,115,116]. Anderson et al. [114] identified the genes regulating CPS biosynthesis and export, showing that disruption of capsule formation diminishes bacterial survival in host environments and heightens serum susceptibility. Capsule locus (KL) analyses of bloodstream isolates further revealed notable genetic diversity, consistent with previous observations [76,120], and classical biochemical and serological studies have distinguished at least 14 CPS antigens [115,116,120]. More recently, Anderson et al. [73] demonstrated that KL variation drives functional differences in virulence: sialylated KL1 and KL2 capsules enhance resistance to macrophage uptake, and KL types differ in organ colonisation during bacteremia, highlighting capsule-type-dependent adaptation among circulating lineages [73].

- **Outer membrane (OM):** The OM is central to Gram-negative biology, supporting adhesion, environmental sensing, host interaction, and cell-to-cell communication [69,100,121,122,123,124,125,126,127,128,129,130,131]. Its defining feature, however, is its permeability barrier, which excludes large or hydrophobic antibiotics and underlies the intrinsic drug resistance of Gram-negative pathogens, an obstacle that continues to impede antibiotic development and drive MDR [41,47,121,127,132,133,134,135].

Among these OMPs, porins facilitate passive uptake of hydrophilic molecules and are broadly classified as specific porins or non-specific porins according to substrate selectivity [136,137,138,139,140,141,142].

Choi and Lee [140] proposed a functional classification of porins into three categories. The first comprises specific uptake channels, such as LamB and YddB. The second includes non-specific porins that predominantly contribute to membrane stability, e.g., OmpA. The third category consists of non-specific porins, including OmpC and OmpF, which perform dual roles by facilitating transport while maintaining membrane integrity.

Hutsul and Worobec reported the identification of OmpC and OmpF orthologues in a clinical isolate of *S. marcescens* (UOC-51) in 1994. These proteins demonstrate approximately 70% sequence identity to their *E. coli* homologs [143,144].

Porin expression is orchestrated by a multifaceted regulatory network involving XylS/AraC-family transcription factors, extracytoplasmic function (ECF) sigma factors, and key two-component systems (EnvZ–OmpR, PhoB/PhoR, CpxAR, PhoPQ, PmrAB, Rcs) [139,144,145,146,147,148].

In *S. marcescens*, OmpF and OmpC expression is governed by the EnvZ–OmpR two-component system (TCS) and the micF small RNA [144,149].

PhoE PhoE, structurally similar to OmpC and OmpF, but differs functionally by exhibiting anion selectivity and a marked preference for phosphate-containing substrates [144,150,151]. Unlike OmpC and OmpF, PhoE is activated by the PhoB/PhoR system, but, like OmpF, it is post-transcriptionally repressed by micF sRNA [144,148,151]. Like OmpC, OmpF, and PhoE, *S. marcescens* LamB forms a homotrimer. However, its structural features are specialised for carbohydrate transport [144,152,153].

*S. marcescens* LamB, which is 78% identical to canonical maltoporin, acts as a broad sugar transporter. Its expression is controlled by the *malEFG* and *malK-lamB-malM* operons, coordinating LamB with ABC transporter-mediated maltodextrin uptake [144,153].

These pathways fine-tune outer-membrane permeability through direct regulation or hierarchical cascades. LPS alterations, envelope stress, and biofilm-associated physiological shifts further influence porin insertion, stability, and overall abundance [139,145,146,147]. Porins such as OmpC, OmpF, OmpN, LamB, and PhoE significantly influence antibiotic susceptibility and frequently act synergistically with enzymatic resistance, target-site alterations, and efflux systems [145,146,154,155].

In this regard, OmpA, OmpC, and OmpF collectively influence outer-membrane integrity and antibiotic permeability, but their roles differ. OmpA functions primarily as a structural anchor through its peptidoglycan-binding C-terminal domain [140,156,157,158,159,160,161,162,163,164,165,166,167,168]. OmpF serves mainly as a high-conductance β-lactam porin with minimal impact on membrane stability. In contrast, OmpC contributes to both β-lactam influx and envelope maintenance by interacting with MlaA in the Mla pathway to remove mislocalized phospholipids, thereby sustaining lipid asymmetry and outer-membrane stability [140,156,157,158,159,160,161,162,163,164,165,166,167,168]. OmpX is a conserved outer membrane protein (first described in *S. marcescens* by Guasch et al. in 1995) that enhances bacterial adhesion, virulence, and adaptability [144,169,170,171].

OmpW, a small Omp family protein, facilitates the transport of hydrophobic compounds, contributing to reduced antimicrobial efficacy [136,172,173]. OmpW mediates host attachment and elicits immune responses to intestinal pathogens. It is immunogenic in *Salmonella*-induced reactive arthritis, celiac disease, pediatric Crohn’s, and oral pathogen-associated inflammatory bowel disease, suggesting a role in mucosal inflammation [172,174,175,176,177,178,179,180,181,182,183,184,185,186,187,188,189,190,191]. OmpW is highly conserved among facultative anaerobes, including major pathogens, suggesting roles in adaptation or virulence [192,193]. Phage LC53 exploits this conservation by using OmpW as its receptor across diverse hosts, and Mahler et al. [194] confirmed OmpW as essential for phage attachment.

- **Extracellular enzymes:** *S. marcescens* employs a broad spectrum of exoenzymes [e.g., proteases, phospholipases, lipases, and nucleases (such as DNases)] to breach host barriers and establish infection. Pathogenic bacteria, including *S. marcescens*, secrete non-cytotoxic extracellular nucleases that play multifaceted roles in disease progression. These enzymes contribute to nutrient acquisition, facilitate immune evasion, modulate biofilm architecture, and enhance HGT [195]. Indeed, nucleases are multifunctional enzymes present across bacterial pathogens like *S. marcescens*, contributing to nutrient acquisition, DNA uptake, and biofilm remodelling. They also promote host invasion through tissue damage, degrade the DNA backbone of neutrophil extracellular traps (NETs) to evade immune clearance, and modulate host immune responses [196].

Proteases, particularly Serralysin (PrtS), are well-recognised for inducing cytotoxicity and inflammation in localised infections such as keratitis [197,198,199,200,201,202,203,204]. Importantly, González et al. [197] reported that isolates from wounds and respiratory sites produce markedly higher protease levels than bloodstream isolates, indicating that protease expression is modulated by the tissue microenvironment to enhance bacterial invasiveness.

The pore-forming hemolysin ShlA is critical to *S. marcescens* pathogenicity. By physically damaging host membranes, ShlA undermines vascular stability, triggers immune responses, and facilitates bacterial entry into tissues. This cascade results in severe localised infections, and the overproduction of ShlA is specifically linked to the emergence of a hypervirulent phenotype [197,198,199,200,201,202,203,204].

The *shlA* and *shlB* loci encode the hemolysin ShlA and its outer-membrane accessory protein ShlB, which together operate through type Vb secretion system (T5bSS) similar to that described in *Yersinia pestis* [201,205]. Indeed, ShlB functions dually as both the secretion system and the activator of the pore-forming toxin ShlA. In the absence of *shlB*, *shlA* accumulates in the periplasm in an inactive, non-hemolytic state, indicating that ShlB is essential for its maturation and functional activation [206].

A distinguishing feature of the *Serratia* system is its activation mechanism: ShlB requires phosphatidylethanolamine (PE) as a cofactor to activate ShlA through a defined conformational shift. ShlA mediates erythrocyte lysis via pore formation, yet at sub-lytic concentrations it induces apoptosis, vacuolization, and cytoskeletal alterations, contributing to tissue damage under physiological conditions [201,205].

Prodigiosin pigment production: Prodigiosin, the red secondary metabolite of *S. marcescens*, has shifted from being viewed as a metabolic byproduct to a molecule with notable ecological and biomedical relevance [207,208]. Although not required for virulence, it contributes to stress resilience, particularly by mitigating oxidative damage, and can act as a competitive factor in polymicrobial settings through its antimicrobial activity [209,210]. Beyond its ecological functions, prodigiosin exhibits broad bioactivity, including potent anticancer, antifungal, antimalarial, and larvicidal effects, along with pronounced immunomodulatory and immunosuppressive properties [208,211]. Prodigiosin, a red tripyrrole pigment produced by *Serratia*, *Vibrio*, and *Streptomyces*, is encoded in *Serratia* spp. by the pigA–pigN biosynthetic cluster [212,213]. Its expression is regulated by a complex network integrating quorum sensing (QS) and two-component systems (TCS), including the SmaI/SmaR and SpnI/SpnR QS circuits and the PigQ/PigW, PhoB/PhoR, RssB/RssA, and EepR/EepS TCS modules [212,213,214,215,216,217]. Additional transcriptional regulators act as activators (PigP, PigT, PigS, PigR, PigV) or repressors (PigX, HexS). Recent genetic studies, including Tn5 transposon mutagenesis, have further expanded this network, identifying rcsA, slyA, and rpoS as important contributors to pigment production, alongside the CpxA/CpxR and EnvZ/OmpR systems as additional positive regulators [212,216,218,219,220,221,222,223]. The results of previous studies show that, disruption of multiple cellular systems strongly impaired prodigiosin synthesis. Insertions in *acrB* and *tolC*, components of the AcrAB–TolC efflux pump, reduced pigment production, and mutations in the Tol-Pal genes *tolR* and *pal* similarly diminished output. Loss of Tai4, a structural element of the type VI secretion system, caused an even greater decline. Additional disruptions in central metabolic genes further highlight the strong linkage between core metabolism and prodigiosin biosynthesis [212].

Lin et al. [205] investigated the role of metalloendopeptidase pitrilysin, encoded by the *ptrA* gene, in *S. marcescens*. Their study demonstrated that the *ptrA* gene not only regulates prodigiosin biosynthesis by activating the *pig* gene cluster but also broadly controls motility (both types of swarming and swimming), biofilm formation, hemolytic activity, and stress tolerance, coordinating key metabolic and virulence traits.

- **Secretion systems (SSs):** The Sec and Tat pathways are the primary conserved routes for protein export across the bacterial cytoplasmic membrane, operating via distinct mechanisms [224,225,226,227]. The Sec pathway transports unfolded proteins through the SecYEG translocase, often with accessory factors in Gram-positive bacteria, and is responsible for secreting many virulence factors [224,226]. In contrast, the Tat pathway exports folded, frequently cofactor-containing proteins via the TatA–TatB–TatC complex (with TatA/B fused in some Gram-positive species). In Gram-positive bacteria, substrates are released extracellularly, whereas in Gram-negative species they remain in the periplasm or are further exported via the type II secretion system (T2SS). The Tat pathway is critical for virulence in several pathogens, including phospholipase C–producing bacteria [227,228,229,230,231,232].

Bacteria use specialised nanomachines to secrete molecules critical for adhesion, virulence, and survival. In Gram-negative species, secretion systems span both membranes (T1SS, T2SS, T3SS, T4SS, T6SS) or only the outer membrane (T5SS, pili, curli). Transport occurs via one-step (cytoplasm to exterior) or two-step (via periplasm) mechanisms. Most systems export unfolded proteins, whereas T2SS, T6SS, and chaperone–usher pathways can secrete folded substrates [42,100,232,233,234,235,236].

- Type I secretion system (T1SS) and RND pumps are tripartite, double-membrane systems. T1SS secrete key virulence and nutrient-acquisition factors, including toxins, adhesins, bacteriocins, and enzymes that support bacterial survival and pathogenicity, while RND pumps export small molecules, including antibiotics. Both use a one-step mechanism, with T1SS relying on an ABC transporter in the inner membrane [233,237,238,239,240,241,242]. Previous studies indicate that *S. marcescens* secretes the hemophore HasA through a T1SS-dependent pathway [224,243]. The Swr QS system in *S. liquefaciens* MG1/*S. marcescens* MG1 regulates both swarming motility and the Lip T1SS, which secretes lipases, metalloproteases, and S-layer proteins [244,245,246].
- Type II secretion systems (T2SSs) in Gram-negative bacteria export folded proteins from the periplasm, receiving substrates delivered by Sec or Tat. They secrete diverse enzymes, e.g., proteases, phospholipases, and toxins, many linked to virulence, and their pseudopilus is evolutionarily related to type IV pili and competence systems [224,247,248,249,250]. The T2SS is widely distributed among Gammaproteobacteria and has been documented in at least 15 genera, including *Serratia*, *Klebsiella*, *Yersinia*, *Acinetobacter*, etc. [20,251].
- Type III secretion (T3SS) systems are multi-component nanomachines that deliver effectors to host cells via a pilus-like structure and a translocon. T3S structural components are encoded in pathogenicity islands (PAIs) within chromosomal or plasmid gene clusters likely acquired through HGT [224,233,252,253,254]. The term also includes flagellar T3SSs, which mainly export structural proteins but can additionally secrete virulence factors. Many bacteria, including *Serratia,* possess multiple T3S systems, including flagellar and translocation-associated types, which function at distinct infection stages [20,224,233,252,253,254]. Translocation-associated systems, while mainly pathogenic, can also support symbiosis and feature appendages—pili in plant pathogens or needles in animal pathogens for protein delivery [224,233,252,255]. Thus, effector proteins are delivered into host cells via the T3SS [20].
- Type IV secretion systems (T4SSs) are the only secretion systems that transfer both DNA and proteins, underpinning plasmid conjugation and contributing to pathogen–host interactions [224,233,256,257]. Widespread “minimised” T4SSs, streamlined derivatives of ancestral conjugative modules, occur across Gram-positive and select Gram-negative bacteria, often linked to MGEs and surface-associated virulence traits [256,257,258,259,260]. *Tn916*-like Integrative and conjugative elements (ICEs), including *Tn6009* identified in *Klebsiella*, *Pseudomonas*, and *Serratia* encode a reduced VirB/VirD4 set yet retain broad host-range mobility. Despite structural variation, most Gram-negative T4SSs conserve a VirB/VirD4 core that mediates conjugation, DNA exchange, and effector translocation [256,257,259,260,261,262]. All in all, T4SS mediates HGT, driving the spread of resistance genes across bacterial populations. It also performs effector delivery and interbacterial antagonism. T4SS has key roles in bacterial pathogenesis and host–pathogen interactions [20,241,263,264].
- Type V secretion systems [(Va–Ve); Va (classical Autotransporters (ATs); Vb (two-partner Secretion); Vc (trimeric AT Adhesins); Vd (a hybrid form of types Va and Vb systems); Ve (inverse ATs)] share a hallmark design in which a single polypeptide encodes both the β-barrel pore and its passenger [265,266,267,268]. This minimalist architecture underlies their self-contained export mechanism, giving rise to the term “autotransporters,” especially for Va, Vc, and Ve [265,266,267,268]. Type Vf represents a recently defined AT subclass restricted to *Helicobacter pylori*, with BapA as its prototypical member [257,268,269,270].

Type Va passengers exhibit considerable functional diversity, particularly in protease activity, and are grouped into three classes: non-serine protease ATs of *Enterobacteriacea* (SPATE) proteases, SPATE-like proteases, and canonical SPATEs [268,271,272]. Comparative studies highlight this breadth; *S. marcescens* encodes the non-SPATE proteases Ssph1/2 (T5aSS) and the ShlA/ShlB cyto-/hemolysin (T5bSS) [267,268,273,274,275,276]; while in *S. liquefaciens*, the lipolytic autotransporter EstA (T5aSS) supports cellular signalling by generating lipids required for second-messenger synthesis [268,277].

- Type VI secretion system (T6SS) is a widespread contractile apparatus in Gram-negative bacteria that underpins interbacterial antagonism, host interactions, and environmental adaptation [278,279]. In *Serratia*, it is key to competitive fitness. Jiang et al. further demonstrated that its broad protein diversity and frequent HGT confer strong evolutionary adaptability [278,279]. Beyond their standard roles, T3SS and T6SS broadly modulate microbial ecology: T3SS can influence microbiota composition, whereas T6SS mediates antifungal activity, metal scavenging, and DNase-dependent interactions in biofilms [280,281,282,283,284,285].

Cummins et al. [286] report that *Serratia* genomes contain three conserved “hotspots” that act as multifunctional defence islands, integrating anti-phage systems, antibacterial elements, and virulence factors. Their analysis revealed four distinct anti-phage strategies, including a newly described TIR-domain system and two loci that repurpose T6SS-associated proteins for defensive roles, underscoring the organism’s modular genomic architecture [286].

- The Type VII secretion system (T7SS), first characterised in *Bacillus* spp., has since been identified in several *Pseudomonas* strains. Unlike typical Gram-negative systems, T7SS (T7SSa/b) in Actinobacteria and Firmicutes mediates virulence, modulates membrane permeability, nutrient (e.g., iron) acquisition, competition, development and niche colonisation [287,288,289,290].
- Type VIII secretion system (T8SS) is known as a Gram-negative bacterial two-step transporter [241]. As aforementioned in the curli-like fibres section, constitution, assembling, and secretion of curli fibres occur via a nucleation–precipitation pathway, recognised as T8SS [42,100,101,108,291,292,293]. As curli-like fibres have been detected in *Serratia* species like *S. marcescens* [20], it seems that T8SSs are detectable in their bacterial cell [101]. In other words, curli biogenesis, classified as the T8SS, is governed by two divergently transcribed operons, *csgBAC* and *csgDEFG* [101].
- Type IX secretion system (T9SS) is unique to Bacteroidetes. T9SS exports virulence factors, degradative enzymes, and motility adhesins—supporting pathogenesis in *Porphyromonas gingivalis* and gliding in *Flavobacterium johnsoniae*. Recent structural studies have clarified its translocon and motor machinery [294,295,296].
- The Type X secretion system (T10SS), originally identified in Gram-negative bacteria, is homologous to phage lysis cassettes and consists of a minimal holin–hydrolase module that mediates controlled protein release [297,298,299,300]. In *S. marcescens* DB10, it enables chitinase export through a pathway distinct from the T2SS. T10SS function requires coordinated holin–hydrolase activity and proceeds through at least two stages, with a possible third step yet to be defined [297,298,299,300].
- The Type XI secretion system (T11SS) is a conserved proteobacterial pathway (e.g., *E. coli*, *A. baumannii*), which functions as a transport channel that moves soluble proteins and lipoproteins across the outer membrane via a dedicated protein complex [301,302,303,304]. The specific molecules it exports are determined by the system’s genetic makeup; known effectors include proteins that interact with transferrin, lactoferrin, factor H, and heme [301,302,303,304].
- **Iron acquisition systems**: For pathogenicity and survival, bacteria require iron acquisition. *S. marcescens* utilises two distinct mechanisms to retrieve iron from heme: the Hem system, which directly extracts the metal, and the Has system, which relies on a hemophore protein to aid in the extraction and transport processes [117,305,306,307].

*S. marcescens* employs two coordinated heme-acquisition pathways adapted to iron limitation. At relatively high heme levels (≥10^−6^ M), uptake is mediated by the TonB-dependent Hem system. Under stringent iron deprivation, the Has pathway is induced, using a secreted haemophore and the dedicated HasR receptor, which operates exclusively with HasB. Together, these systems enable efficient haem scavenging across a wide range of environmental conditions [307].

Pathogens such as *S. marcescens* and *P. aeruginosa* utilize the ATP-independent HasA hemophore system for heme uptake, involving the HasA hemophore, the HasR outer-membrane receptor, and the TonB-like protein HasB [308,309,310,311,312,313,314,315,316]. Unlike TonB, HasB is dedicated exclusively to HasR, reflecting the high receptor specificity of this system and its adaptation for efficient heme scavenging in Gram-negative bacteria [308,309,310,311,312,313,314,315,316]. In *S. marcescens*, iron uptake is driven by the ExbB–ExbD–TonB complex. ExbB interacts with HasB via its periplasmic extension, while membrane residues coordinate interactions with both TonB and HasB, collectively supporting inner-membrane iron acquisition [317].

Genomic studies confirm that *S. marcescens* and *S. plymuthica* produce two iron-chelating siderophores: serratiochelin (a hybrid compound composed of enterobactin and vibriobactin) and chrysobactin (a low-affinity siderophore). When iron is scarce, these molecules bind to Fe^3+^ ions and transport them into the cell, a process essential for the bacteria’s survival and growth in low-iron environments [306,318,319,320,321].

- **Quorum sensing (QS) and regulation**: In contrast to eukaryotes, which rely on hormone-mediated signalling, prokaryotic communication is largely governed by QS mechanisms [322]. Bacteria display coordinated social behaviours via QS, a cell-to-cell communication mechanism. QS relies on autoinducers (AIs) (chemical signals produced by pathogens) that trigger collective procedures once a threshold concentration is reached, enabling bacteria to perform tasks unattainable by individual cells [323,324]. AHLs/AIs constitute the principal QS signals in Gram-negative bacteria, regulating density-dependent gene expression and collective behaviours, including biofilm formation [20,323,324,325,326]. Although primarily involved in intra-species communication, they can also facilitate interspecies signalling. The best-characterised framework for AHL-dependent QS is the *Vibrio cholerae, V. harveyi, V. fischeri* and *Myxococcus xanthus* LuxI/LuxR system. LuxI produces the autoinducer, which binds to the cytoplasmic receptor LuxR. This interaction induces a conformational change in LuxR, allowing it to bind DNA and regulate target gene expression [20,323,327].

*Serratia* species exhibit unusually diverse QS systems despite the broad conservation of LuxI/LuxR homologues. In *Serratia*, multiple LuxI/LuxR-type systems have been identified. Examples include SmaI/SmaR (*Serratia* sp. ATCC 39006), SpnI/SpnR (*S. marcescens* SS-1), SwrI/SwrR (*S. liquefaciens* MG1/*S. marcesens* MG1 [246]), and SprI/SprR (*S. proteamaculans*) [214,325,328,329,330,331,332].

In *Serratia* spp. ATCC 39006, the SmaI/SmaR system is involved in the production of carbapenem and prodigiosin [88,330]. In *S. marcescens* strain 12, the SmaI/SmaR QS system governs multiple coordinated procedures, including swarming motility, hemolytic activity, and biofilm development, among other virulence-associated traits [88,333]. The SwrI/SwrR QS system in *S. liquefaciens* MG1/*S. marcescens* MG1 [246] has been shown to coordinate several key phenotypes. This regulatory pair promotes swarming motility, drives the production of serrawettin, extracellular proteases, and S-layer proteins, and enhances both biofilm formation and 2,3-butanediol fermentation [88,329].

In *S. marcescens* SS-1, SpnI produces four AHLs, and SpnR—a repressor relieved by 3OC6-HSL—controls sliding motility, the production of biosurfactant, prodigiosin, rhamnolipid, and nuclease. The *spnI/spnR* module, located on a mobile *Tn3* transposon, confers AI production and substantially alters its metabolic profile [88,214,325,328,329,330,331,332].

As aforementioned, QS regulates the expression of genes associated with biofilm formation, biosurfactant synthesis, virulence factor production, pathogenicity, infection, and antibiotic resistance in *S. marcescens* [90,325,334,335]. The resilience of *S. marcescens* stems primarily from its dual defence strategies. β-lactamase production provides an effective enzymatic barrier against β-lactam antibiotics, while robust biofilm formation supplies a physical refuge. The biofilm’s extracellular polymeric substance (EPS) creates a protected niche that facilitates cell-to-cell communication, genetic exchange, and coordinated behaviour, all while limiting access by host immune factors [336,337,338]. Normally, the composition of EPS comprises protein molecules, polysaccharides, or extracellular/environmental DNA (eDNA). The EPS matrix functions as a structural and protective biopolymer, shielding cells from antimicrobial agents and host immunity while promoting aggregation [336,337,338]. It also enhances desiccation resistance, aids nutrient retention, and serves as a carbon source. Together, these mechanisms underscore the pathogen’s sophisticated adaptive capacity and clinical persistence [336,337,338]. Evidence suggests that QS is a key mechanism facilitating communication within the gut microbiota and between microbiota and host cells [129,183,184,257,322,325,339,340,341].

While QS regulates biosurfactant production, biosurfactants in turn modulate QS signalling and QS-dependent processes, including biofilm formation, motility, and pathogenicity [90,342,343,344]. Originating from bacteria, fungi, and yeasts, biosurfactants are secondary metabolites that function either within the cell membrane or as secreted extracellular substances. They facilitate cellular attachment and dispersal by creating thin films, while also orchestrating critical biological processes such as motility, antagonism, and intercellular signalling [90]. Biosurfactants are classified by composition and molecular weight, the low-mass types and the high-mass types [90].

QS inhibition offers a novel antibacterial approach by disrupting microbial communication and virulence without imposing strong selective pressure [345,346]. QS inhibition limits resistance development and attenuates bacterial virulence by targeting communication instead of essential processes [345,347].

## 4. Antimicrobial Resistance (AMR) Mechanisms in CRSM

*Serratia* spp. carry a broad set of resistance determinants against multiple antibiotic classes. These arise from intrinsic, chromosome-encoded mechanisms as well as acquired mutations or HGT, mobilised by plasmids, integrons, ISs, transposons, and GIs that facilitate rapid dissemination of resistance [43,91,348]. The diversity, mobility, and mechanisms of many MGEs remain largely unknown [349]. Over half of plasmids lack the machinery for autonomous transfer and likely depend on alternate routes such as transduction, transformation, conjugation, vesiduction or helper plasmids [43,349,350,351,352,353]. Most putative genomic insertions also remain uncharacterized, highlighting major gaps in understanding how MGEs originate, spread, and shape microbial evolution [43,349,350,351,352,353]. MGEs disseminate resistance by inserting into bacterial chromosomes, where they may become permanently fixed or remain excisable and mobile. This dynamic is regulated by integrases encoded by the element itself, or supplied by the host, which mediate precise excision from the genome and thereby enable subsequent HGT [348,354].

Bacteria are evolving into “superbugs” that survive standard treatments by using four main mechanisms: preventing drug entry, modifying drug targets, neutralising drugs chemically, or expelling them via efflux pumps [37,43,45,46,91,348]. The main factor of this procedure is HGT, a process that allows bacteria to rapidly share resistance genes, often clustered together across different species. This enables bacteria to receive ARGs in a single event, making these resilient strains incredibly difficult to eliminate [348,355,356].

The overuse/misuse/self-medication of antibiotics has triggered a global health emergency known as AMR. Indeed, the escalation of the AMR phenomenon is driven by intertwined pressures, including antibiotic overuse/misuse/self-medication and inadequate sanitation in human settings, extensive antibiotic application in agriculture and aquaculture, environmental contamination, and ecological disruptions that promote wildlife-associated transmission [348,355,356,357].

*Serratia* species display pronounced AMR driven by the combined action of intrinsic, acquired, and adaptive mechanisms [26].

*Serratia* spp. exhibit strong intrinsic resistance, widely via efflux pumps, against β-lactams, polypeptides, macrolides and quinolones. Yet, they typically remain susceptible to trimethoprim and sulfonamides, likely because resistance to these agents imposes a high fitness cost or has been rarely selected [33,36,91,238,358,359,360,361]. Although plasmid-mediated resistance occurs sporadically, it remains uncommon. Targeting efflux regulation may offer a strategy for next-generation treatments, while advances in genome sequencing accelerate the detection of resistance determinants [33,36,91,238,358,359,360,361]. Multidrug-resistance is widespread in *Serratia*, largely due to its extensive efflux pump repertoire, complicating treatment. However, tailoring treatment to the strain’s genomic and resistance profile enables clinicians to avoid ineffective agents and improve therapeutic outcomes [91,238].

Efflux pumps are transmembrane transporters that expel toxic compounds (including antibiotics, detergents, and heavy metals) from the bacterial cytoplasm, thereby supporting intrinsic tolerance to diverse stressors. Mutational changes can further modify pump activity or specificity, contributing to acquired resistance [26,362,363,364,365,366]. Although efflux pumps are widely recognised for their role in antibiotic export, their functional scope is considerably broader. They participate in key normal processes, including the removal of endogenous metabolites, transport of siderophores, and regulation of QS signals [366,367,368,369,370]. Bacterial antimicrobial efflux systems are categorized into six major superfamilies, defined by their amino acid sequences, topologies, structures and energetic requirements, including: (a) the ATP-Binding Cassette (ABC) and (b) Major Facilitator Superfamilies (MFS), as well as (c) the Resistance–Nodulation–Cell Division (RND), (d) Drug/Metabolite Transporter (DMT), e.g., Small Multidrug Resistance (SMR) family, (e) Multidrug and Toxic Compound Extrusion (MATE), (f) Proteobacterial Antimicrobial Compound Efflux (PACE), and an additional family, the p-Aminobenzoyl-glutamate Transporter (AbgT) [26,44,367,371].

Genomic analysis of *S. marcescens* has identified efflux pumps from five of the seven major families (ABC, MATE, MFS, SMR, and RND), with the PACE family being the only one absent. While pumps from the ABC, RND, DMT (SMR), and MFS families are known to contribute to multidrug-resistance, the specific function of the MATE family homologs in this species remains uncharacterized [26,76,91,371].

The DMT superfamily, present in all life forms, includes over 30 families that transport metabolites and drugs. While some are compact four-helix pumps, others evolved into complex ten-helix systems. The SMR family exemplifies this diversity, comprising small four-helix pumps that function independently [371,372,373,374,375].

ABC transporters form a universally conserved superfamily and represent the most prevalent transport systems across all life domains [371,376,377,378]. Encompassing nearly 100 families, they translocate a broad spectrum of substrates, including nutrients, ions, drugs, and xenobiotics. In bacteria, they function primarily in substrate uptake, while efflux activity is widely conserved in both prokaryotes and eukaryotes [371,376,377,378].

In *S. marcescens*, the MacAB efflux pump is vital for bacterial viability. Although its removal does not alter resistance to macrolides, it significantly heightens sensitivity to colistin and aminoglycosides [26,367,371,379]. Furthermore, MacAB is required for oxidative stress response, motility, and biofilm formation. Therefore, inhibiting this pump offers a viable approach to reduce both the virulence and antibiotic resistance of the organism [26,367,371,379].

In *S. marcescens*, RND pumps drive key multidrug resistance. SdeXY mediates intrinsic resistance, especially to tetracyclines and fluoroquinolones. SdeAB adds broader MDR, while SdeCDE is limited to novobiocin [26,363,371,380,381,382,383,384]. In strain Db10, SdeGH and SdePQ-OmsA export diverse substrates, whereas SdeIJ is specific for benzalkonium chloride. Collectively, these systems equip the bacterium to withstand multiple antimicrobials [26,363,371,380,381,382,383,384].

In *S. marcescens*, SMR transporters are small four-helix dimers that route toxins to the periplasm, yet their role in antibiotic resistance remains unclear [26,371,385,386,387]. SsmE expels ethidium bromide, acriflavine, and norfloxacin, whereas the newer pumps SsmD and SsmK show no clear link to resistance, leaving their functional relevance unresolved [26,371,385,386,387].

In *S. marcescens*, MFS transporters—typically 12–14-helix exporters—support AMR by removing diverse toxic compounds [371,388,389,390,391]. SmfY expels norfloxacin and cationic agents, SmvA confers resistance to biocides such as chlorhexidine, and TetA mediates tetracycline resistance under TetR control. Additional uncharacterized MFS members likely broaden the species’ multidrug resistance capacity [371,388,389,390,391].

Aminoglycoside resistance is mainly conferred due to modifying enzymes, but low gene expression generally preserves clinical susceptibility [26,34,36,91,392,393,394,395,396,397,398,399,400].

Fluoroquinolone resistance can arise through two primary mechanisms: mutations in DNA gyrase or topoisomerase IV, or the acquisition of plasmid-mediated determinants like *qnr*, *qepA*, *aac(6′)-Ib-cr*, and *oqxAB*. The *aac(6′)-Ib-cr* variant is particularly common in strains resistant to both fluoroquinolones and aminoglycosides, a pattern consistent with prior studies connecting the gene to reduced susceptibility in both antibiotic families [40,43,395,401,402,403].

*S. marcescens* exhibits intrinsic resistance to macrolides and cationic antimicrobial peptides (CAPs) such as polymyxins, driven by low outer-membrane permeability and *arnBCADTEF*-mediated LPS modifications, although polymyxins serve as a key last-resort treatment option against Gram-negative superbugs [26,34,45,91,404,405,406]. The PhoP/PhoQ and PmrA/PmrB systems—modulated by MgrB—induce *arn* expression in response to environmental cues, promote polymyxin and colistin resistance across *Serratia* species [26,34,91,404,405,406,407]. Beyond CAPs, *Serratia* spp. shows broad intrinsic resistance, frequently carrying *bacA* (bacitracin), *hslJ* (novobiocin), *fosA* (fosfomycin), *cat* (chloramphenicol), and occasionally *vanX* (vancomycin) on the chromosome [26,91,408,409].

B-Lactam resistance in *S. marcescens* is largely mediated by β-lactamases, including AmpC enzymes, ESBLs, and carbapenemases. The chromosomal AmpC is universally present and inducible, conferring resistance to penicillins and third-generation cephalosporins; carbapenem resistance can emerge when AmpC overproduction coincides with porin loss [26,34,36,91,392,393,394,395,396,397,398,399,400]. Derepression is less frequent in *S. marcescens* than in other *Enterobacterales*, though mutations can expand AmpC activity. Carbapenem resistance may also arise from the rare, chromosomal SME carbapenemase [26,34,36,91,392,393,394,395,396,397,398,399,400]. As mentioned, *S. marcescens* resistance is largely driven by a broad repertoire of β-lactamases, including acquired ESBLs and carbapenemases as well as their intrinsic, inducible AmpC cephalosporinase. Class A enzymes (such as SME and KPC) and class B MBLs are widely reported globally, while class D variants (like OXA), though still less prevalent, are becoming increasingly detected [410,411,412,413,414]. *S. marcescens* is particularly concerning because it is intrinsically resistant to colistin. Once it acquires carbapenem resistance, it becomes impervious to both major last-line treatments, effectively eliminating viable treatment options. This dual resistance is associated with markedly elevated treatment failure. Mortality rates approaching 55% have been reported in critically ill patients with CRSM and dual resistance mechanisms in observational studies and case reports, although these estimates vary depending on patient population and study design [415,416,417,418,419].

## 5. CRSM and the Impact of Transposable Elements (TEs)

Managing the ‘CES’ pathogens (*Citrobacter* spp., *Enterobacter* spp., and *Serratia* spp.) is challenging because they carry intrinsic resistance to penicillins, cephamycins, and early-generation cephalosporins via AmpC β-lactamases [38,420,421,422,423,424,425]. *Serratia* spp. add inherent resistance to nitrofurantoin, doxycycline, colistin, macrolides and most of the aminoglycosides. Although classified under the ‘SPICE’ group for their clinical relevance, rising MDR strains with plasmid-borne ESBLs or carbapenemases further constrain treatment, often necessitating expensive last-line antimicrobial agents such as tigecycline, ceftazidime–avibactam, meropenem-vaborbactam or imipenem-relebactam. ‘SPICE’ term is an acronym for *Serratia*, *Pseudomonas*, indole-positive *Proteus*, *Citrobacter*, and *Enterobacter* [38,47,91,135,420,421,422,423,424,425,426,427,428,429,430]. Furthermore, as mentioned before, *Serratia* spp., similar to other ESCPM organisms, is intrinsically resistant to many β-lactams via chromosomal AmpC. The rise in plasmid-borne ESBLs and carbapenemases has compounded this, as these SBLs further limit effective therapy [32,33,34,35,36,37,47,135].

Although carbapenemase-production is the primary factor of resistance in Gram-negative bacteria like *Enterobacteriaceae*, decreased OMP permeability combined with the overexpression of AmpC or ESBL enzymes can also lead to reduced susceptibility [431]. Carbapenemases—classified as Class A (e.g., KPC), Class B (e.g., VIM, NDM, IMP), and Class D (e.g., OXA variants such as OXA-48)—function by hydrolysing carbapenems and other β-lactams [36,37,40,41,43,394,415,432,433,434]. Their rapid dissemination is facilitated by large conjugative plasmids. Conversely, resistance arising from OMP loss and AmpC/ESBL overproduction is not plasmid-borne; rather, it spreads primarily through clonal expansion [431,433,434,435].

Plasmid-mediated β-lactamases spread readily in *S. marcescens* through HGT, with Amber’s Class A ESBLs, KPCs (e.g., CTX-M, SHV) and Class D OXA variants (e.g., OXA-48) underpinning most clinically relevant resistance [36,43,395,415,431,436,437,438,439].

KPC-producing strains, often acquired in hospital settings, are now globally distributed (such as Brazil, China, Greece, Italy, United States), spreading through different types of plasmids, including IncX6-like and IncX8 plasmids [401,417,440,441,442,443,444,445,446,447].

Bolourchi et al. [415] identified the *bla*_OXA-48a_ gene as the cause of carbapenem resistance in four Iranian *S. marcescens* isolates. Their investigation revealed that the gene was plasmid-borne, residing on an IncMCR incompatibility group plasmid [415].

Pérez-Viso et al. [448] investigated carbapenemase-producing *S. marcescens* in a Madrid hospital (2016–2018). Most isolates carried VIM-1, fewer produced OXA-48, and they found out seven lineages dominated by two clones [448]. Nearly all strains possessed an IncL plasmid related to IncL-pOXA-48a, with two variants identified: IncL-pVIM-1, containing an MDR integron, and IncL-pOXA-48, encoding only bla_OXA-48_–*Tn1999* [448]. The results obtained by Pérez-Viso et al. indicate ongoing CPSM transmission across care settings and emphasise the role of *S. marcescens* in local carbapenemase-producing *Enterobacterales* (CPE) spread beyond the usual *Enterobacterales* reservoirs [448].

Pangenome analyses reveal lineage-specific patterns: Sm7 typically carries *bla*_KPC-2_, Sm3 shows high plasmid diversity, and lineages Sm9 and Sm12 harbour the *mcr-9* colistin-resistance gene [1,26]. The *bla*_KPC-2_ gene is typically flanked by a *Tn3* resolvase and ISKpn27 upstream, and an inverted sequence downstream [1,442]. A study classifying *S. marcescens* into 12 distinct lineages reveals significant differences in their distribution and resistance profiles [1]. While environmental samples are concentrated in the Sm1, Sm4, and Sm10 groups, the Sm5, Sm6, and Sm7 groups are exclusively human-associated and show signs of niche specialisation [1]. The most concerning resistance profiles are found in Sm7 and Sm9; Sm7 is linked to plasmids carrying the *bla*_KPC-2_ gene, while the human-restricted Sm9 displays the highest plasmid diversity and burden, including *bla*_NDM-1_ [1]. These findings highlight the urgent need for genomic surveillance to monitor carbapenemase-producing *S. marcescens* [1].

Matteoli et al. showed that the broad global distribution of most *S. marcescens* lineages reflects deep evolutionary roots, whereas the regional restriction of Sm2 in Asia and Sm6 in Europe suggests more recent emergence. The predominance of the most divergent lineages (Sm1 and Sm10) in environmental sources further supports an evolutionary shift from a natural reservoir to a human-associated pathogen [1].

In 2011, a neonatal ward in Argentina reported its first documented nosocomial outbreak of MBL-producing *Enterobacteriaceae* [449]. Nastro et al. [449] identified seven MDR *S. marcescens* isolates, all belonging to a single clone and harbouring a class I integron carrying *bla*_VIM-16_.

Carbapenem resistance in *S. marcescens* is uncommon, arising mainly from porin loss or MBLs. Chromosomal class A carbapenemases (SME, NMC-A, IMI-1) are clavulanate-susceptible, while plasmid-borne class B MBLs (IMP, VIM, NDM) represent the greatest clinical threat, hydrolysing most β-lactams, evading modern inhibitors, and spreading via integrons [410,420,450,451,452]. IMP-1, the first transferable carbapenemase identified in *S. marcescens*, isolated from a Japanese urinary tract infection case in 1991, illustrates the remarkable mobility of these resistance determinants [410,420,450,451,452]. Although VIM enzymes are largely restricted to *Pseudomonas*, plasmid-mediated MBLs have caused outbreaks worldwide, with recent isolates co-carrying *bla*_IMP_ and mcr-9 on conjugative plasmids, highlighting the expanding dissemination and clinical impact of mobile resistance in this species [1,47,135,450,451,453].

Zhong et al. [454] reported that *S. marcescens* YL4 carries chromosomal resistance genes (*bla*_SRT-1_, *aac(6′)-Ic*, *tet(41)*) and plasmid-borne determinants, including *mcr-9*, which can reduce colistin susceptibility and potentially spread to other bacteria via HGT. The megaplasmid pYL4.1 (IncHI2/2A) shares high similarity with *bla*_IMP-26_-harbouring plasmids from *E. cloacae* [455] and *E. hormaechei* [456], suggesting evolutionary links [454]. *IS26* and *Tn3* mediate plasmid rearrangements and gene amplification, while class I integrons facilitate mobilisation of resistance cassettes [43,457]. These MGEs drive dissemination of multidrug and carbapenem resistance among *Enterobacterales*, posing significant clinical challenges [454].

The *IS26* family—which includes elements such as *IS257/IS431*, *ISSau10*, *IS1216*, *IS1006*, and *IS1008*—plays a major role in disseminating antibiotic resistance across diverse pathogens, yet *IS26* remains the best-characterised and most intensively studied member [457]. In Gram-negative bacteria, *IS26* is a dominant MGE that accelerates the spread of resistance by assembling and mobilising gene clusters on plasmids [457]. It forms pseudo-compound transposon and translocatable units, enabling targeted cointegration events that restructure plasmids and amplify AMR loci [457]. *IS26*-family elements also modulate adjacent gene activity and promote extensive plasmid remodelling [457]. These dynamics are central to resistance dissemination in diverse species, including *S. marcescens*, where *IS26*-driven rearrangements enhance plasmid adaptability and transfer [457]. Due to this knowledge, several ISs and transposons—most notably *ISEcp1*, *IS26*, *IS903*, *IS1380*, and *Tn3*—play central roles in capturing and mobilising ESBL-associated genes [458].

Huang et al. characterised an IMP-4–producing, CRSM (S378) from an asymptomatic UTI in Sichuan, China. WGS identified five resistance genes—*bla*_IMP-4_, *bla*_SRT-2_, *aac(6′)-Ic*, *qnrS1*, and *tet(41)*. Conjugation assays confirmed transfer of the *bla*_IMP-4_ plasmid pS378P, an IncN replicon (GC 50%) nearly identical to pP378-IMP [459].

Ghaith et al. [460] reported a NICU outbreak of *S. marcescens* at Cairo University Hospital over a five-month period in 2015. The predominant MBL determinants were *bla*_IMP-4_ (42.5%) and *bla*_VIM-2_ (37.5%) [460]. This investigation provided the first evidence of IMP-4– and VIM-2–producing *S. marcescens* causing bacteremia in this ICU [459,460].

*Bla*_IMP-4_, the most frequently reported IMP variant, is commonly embedded within class I integrons and disseminated via several types of vehicles, including IncHI2, IncL/M, IncA/C, and IncN plasmids [419,461].

As aforementioned, carbapenem resistance in *Enterobacterales* is primarily mediated by five major carbapenemase families—KPC (Class A); IMP, NDM, and VIM (Class B); and OXA-48-like (Class D). Because these enzymes are usually encoded on mobile vehicles of plasmids, they disseminate efficiently through HGT. Such plasmids often harbour additional resistance determinants, including those conferring aminoglycoside and quinolone resistance, further limiting therapeutic options for CPE [419,462,463,464]. Several plasmid families are pivotal in carbapenemase dissemination within *Enterobacteriaceae*, with IncF, IncL/M, IncA/C, and IncX being the most prevalent. In this regard, IncF plasmids are globally widespread and commonly carry KPC, NDM, and ESBLs such as CTX-M-15; whereas IncX plasmids, particularly IncX3, are major vectors for NDM spread. In addition, IncL/M plasmids primarily transmit NDM and OXA-48, while IncA/C plasmids facilitate the dissemination of NDM, VIM, KPC, and CMY-type cephalosporinases [419,465,466].

Formerly grouped together, IncL and IncM plasmids are now recognised as distinct. IncL plasmids drive the global dissemination of OXA-48, whereas IncM2 plasmids transmit NDM-1 and carry multiple resistance genes conferring broad resistance to cephalosporins, carbapenems, aminoglycosides, trimethoprim, sulfonamides, and fosfomycin [467,468].

Sabtcheva et al. [419] analysed carbapenemases in the ESCPM bacterial group, including *S. marcescens*, finding VIM-4 chromosomally encoded in *S. marcescens*. Furthermore, VIM predominated in bacterial strains of *Providencia stuartii* and *S. marcescens* [419]. Furthermore, they found that among Bulgarian CP-ESCPM isolates, *S. marcescens* carried plasmid-borne carbapenemases: OXA-48 on IncL, NDM-1 on IncM2 and IncX3, and VIM-4 on IncM2, underscoring its role as a reservoir of transferable resistance [419].

MGEs drive plasmid and bacterial evolution by reshaping plasmid architecture through transposons and ISs, sometimes improving host compatibility, as seen with *IS26*-mediated deletions in *E. coli*. *IS26* spreads resistance via *IS26*-flanked cointegrates or translocatable units, while *Tn3* transposons mobilise resistance genes through a copy-and-paste mechanism, promoting interbacterial transfer [40,41,43,44,454,469,470].

Zhu et al. [437] analysed 3769 *Serratia* genomes, enrolled between 1823 and 2024 from 65 countries, expanding the genus’ diversity with 14 new species and 809 additional sequence types (STs). *S. sarumanii*, *S. nevei*, and *S. marcescens* were most common, with ST367 and ST324 dominating [437]. Moreover, carbapenem resistance appeared in 26.6% of isolates and was driven by 34 genes, mainly *bla*_KPC-2_, *bla*_SPR-1_, and *bla*_KPC-3_. Resistance emerged after 2011 and now spans 46 countries [437]. Zhu et al. [437] identified 94 putative transmission clusters, including major ST324 spread in the USA. MDR was widespread (61.6%), supported by numerous mobile elements—248 IS types and 61 plasmid replicons—highlighting their central role in disseminating resistance determinants [437]. Zhu et al. [437] reported that carbapenemase-producing *Serratia* carried 248 ISs (*ISSen4*, *IS26*, *IS903B*) and 61 plasmids (ColRNAI_1, Col440II_1, IncFIB(K)_1_Kpn3), displaying gene-specific MGE patterns: *ISs* showed gene-specific correlations: *ISEc33/ISEc36* negatively correlated with *bla*_IMP-1_*, IS1 × 3/ISPa26* positively with *bla*_IMP-13_*; ISAba125* negatively with *bla*_NDM-1_, while other ISs linked positively to NDM, OXA, and VIM variants. They [437] indicated that key plasmids were associated with *bla*_IMP_*, bla*_KPC_*, bla*_NDM_*, bla*_OXA_*, and bla*_VIM_. No IS or transposons correlated with *bla*_SME_, underscoring a complex, element-specific mobilome underpinning resistance [437].

Aracil-Gisbert et al. [32] analysed ICU sinks (2019–2021) and clinical (2003–2019) *Serratia* isolates with 165 genomes, identifying six clades: 1A (*S. nematodiphila*), 1B (*S. marcescens*), 2A (*S. bockelmannii*), 2B (*S. ureilytica*), 3 (*S. marcescens/S. nevei*), and 4A–4B (*S. nevei*), with clades 3–4 predominating. Aracil-Gisbert et al. [32] showed that persistent sink lineages—4A (ST92, ST490) and 4B (ST424) were clonally linked to outbreak strains carrying *bla*_VIM-1_ or *bla*_OXA-48_ on IncL/pB77-CPsm plasmids [448], highlighting sinks as reservoirs for carbapenemase plasmids and their transmission into patients. Among 190 plasmids, 142 clustered into 10 plasmid groups: PG1 (IncL/L-M), PG2, PG3, PG9, PG11, PG12 (Col), PG4 (IncI1, clinical only), PG7–PG8 (IncF-like), and PG10/others (untyped) [32]. PG1 included IncL plasmids with a class I integron, found in *Serratia* clades 3–4 and in *K. pneumoniae*, *K. variicola*, *C. cronae*, and *E. roggenkampii*; variants included a truncated IncL and an IncM1 tetA/tetR plasmid in sinks. PG8 comprised IncF-like plasmids in sinks and patients (subclade 2B, clade 3, subclade 4B) resembling p87710 and carrying fimbrial genes, PG7 matched clinical pE28_003, multiple Col PGs occurred in clades 3–4, and PG4/IncI1 plasmids were confined to clinical isolates [32].

In another study, Mauffrey et al. [471] documented two CPE outbreaks involving patients and contaminated sink traps. Among 57 *K. pneumoniae* and *S. marcescens* isolates, genomic analysis revealed 22 plasmid clusters including *bla*_NDM-1_ and *bla*_KPC-2_, shared between clinical and environmental sources [471]. Their findings highlight sink traps as reservoirs enabling plasmid-mediated resistance spread beyond clonal transmission [471].

Although IncU and IncX3 plasmids play a pivotal role in spreading the *bla*_KPC_ and *bla*_NDM_ resistance genes across various *Serratia* species [472,473,474], the specific relationships between plasmid types, antibiotic resistance genes, and their bacterial hosts remain poorly characterised [474].

Liu et al. [474] analysed 2632 *S. marcescens* genomes from 49 regions and found 350 (13.3%) carrying *bla*_KPC_ and/or *bla*_NDM_, predominantly clinical isolates. Prevalence reached 100% in Ecuador, Chile, Ethiopia, and Iraq, with notable rates in Romania (70%), Bangladesh (52.38%), and Colombia (50%) [474]. The USA (20.78%) and China (45.45%) contributed the largest numbers. Overall, 284 strains carried *bla*_KPC_, 64 *bla*_NDM_, and two strains carried both genes, with KPC-2 and NDM-1 the most common [474]. Phylogenomics revealed five species and 126 ARGs, enriched in wastewater isolates. Species-specific distributions of *Tn4401*/NTEKPC and plasmid types—including IncL/M, IncU, and IncX3—highlighted distinct mobility patterns [474]. Moreover, in the study conducted by Liu et al. [474], multiple ESBL genes were detected, and 283 strains carried both ESBLs and carbapenemase genes (*bla*_KPC_ and/or *bla*_NDM_). A species-layered pattern emerged [474]: *S. nevei* showed the broadest resistance profile, dominated by *bla*_KPC-2_, *bla*_TEM-1_, *bla*_OXA-9_, and *bla*_SHV-30_. *S. sarumanii* was characterised by prominent *bla*_OXA-1_, *bla*_CTX-M-14_, and *bla*_TEM-1_, supplemented by *bla*_KPC-2_ and *bla*_KPC-3_. In contrast, *S. ureilytica* carried fewer determinants overall but consistently retained key genes such as *bla*_TEM-1_ [474].

In a study performed by Xu et al. [475], they showed that CRSM posed major clinical challenges. In 14 isolates, whole-genome and expression analyses revealed three resistance pathways: acquisition of *bla*_KPC_, enhanced *bla*_KPC_ expression, or loss of an outer-membrane protein with elevated *bla*_CTX-M-14_ [475]. These findings indicate diverse in vivo evolutionary routes (e.g., transformation) rather than clonal replacement [475].

Zheng et al. [476] found that, beyond *bla*_KPC-2_, 121 *S. marcescens* genomes carried 52 additional resistance genes across 10 antimicrobial classes. Among 14 newly sequenced isolates, key determinants included *aac(3)-IId*, *aac(6′)-Ic*, *bla*_LAP-2_, *bla*_SRT-1_, and *tet(41)*. Resistance patterns varied by cluster: β-lactam and aminoglycoside genes were most diverse, with *aac(6′)-Ic* (99.2%), *bla*_SRT-1_ (79.3%), and *qnrS1* (72.7%) predominating [476]. *bla*_LAP-2_ and *bla*_CTX-M-14_ were geographically restricted to Chinese isolates. Three strains carried both *bla*_KPC-2_ and *bla*_NDM-1_ [476]. Plasmid typing revealed IncFIIK, IncFII, IncM, and IncR groups, while K17 and K1030 lacked known plasmids. Due to this knowledge, Zheng et al. [476] identified a novel plasmid lineage, pK17-KPC/pK1030-KPC, carrying *bla*_KPC-2_ and enabling HGT, providing crucial evidence for controlling CRSM.

Wang et al. [477] performed a genomic analysis of a *S. marcescens* isolate (L4843) from a faecal sample in China, which harboured both *bla*_KPC-2_ and *bla*_CTX-M-14_ resistance genes. WGS and conjugation assays revealed that *bla*_KPC-2_ was located on a transferable plasmid characterised by complex ISs and transposons [477]. This study marks the first report of a faecal isolate in China carrying both resistance genes, underscoring the critical need for enhanced surveillance of MGEs [477].

## 6. Conclusions

*S. marcescens* has transitioned from a historically intriguing microorganism to a clinically significant opportunistic pathogen shaped by remarkable genomic plasticity and an expansive accessory genome. Its ability to acquire and disseminate mobile genetic elements—including plasmids, integrons, transposons, bacteriophages, and extracellular vesicles—drives the rapid evolution of its resistome and virulome. The convergence of intrinsic resistance, potent efflux systems, and the escalating spread of ESBLs and carbapenemases has positioned *S. marcescens* among the most challenging Gram-negative threats, particularly in ICU settings. Phylogenomic studies reveal distinct lineages with niche-specific adaptations, underscoring the dynamic evolutionary pathways that complicate surveillance and outbreak control.

Given the organism’s increasing multidrug- and occasionally pan-drug-resistance, effective containment will require integrated genomic surveillance, high-resolution epidemiology, and targeted antimicrobial stewardship. A deeper understanding of its mobile genetic landscape and regulatory networks is essential for developing future treatment and infection control strategies.

## Data Availability

No new data were created or analysed in this study. Data sharing is not applicable to this article.
